# Evaluation of factors affecting epidermal growth factor receptor tyrosine kinase inhibitor-induced hepatotoxicity in Japanese patients with non-small cell lung cancer: a two-center retrospective study

**DOI:** 10.1186/s40780-022-00258-7

**Published:** 2022-12-01

**Authors:** Hirofumi Nagai, Tsutomu Shimada, Yoshimitsu Takahashi, Mikako Nishikawa, Hiroyuki Tozuka, Yasuto Yamamoto, Osamu Niwa, Yutaka Takahara, Arimi Fujita, Katsuhiko Nagase, Kazuo Kasahara, Seiji Yano, Yoshimichi Sai

**Affiliations:** 1 Department of Clinical Pharmacokinetics, Graduate School of Medical Sciences, Kanazawa University, 13-1 Takara-machi, Kanazawa, Ishikawa 920-8641 Japan; 2 Department of Hospital Pharmacy, University Hospital, Kanazawa Medical University, 1-1 Daigaku, Uchinada-machi, Kahoku-gun, Ishikawa 920-0293 Japan; 3 Department of Hospital Pharmacy, University Hospital, Kanazawa University, 13-1 Takara-machi, Kanazawa, Ishikawa 920-8641 Japan; 4 Research Support Center, Medical Research Institute, Kanazawa Medical University, 1-1 Daigaku, Uchinada-machi, Kahoku-gun, Ishikawa 920-0293 Japan; 5 Department of Respiratory Medicine, Kanazawa Medical University, 1-1 Daigaku, Uchinada-machi, Kahoku-gun, Ishikawa 920-0293 Japan; 6 Innovative Clinical Research Center, University Hospital, Kanazawa University, 13-1 Takara-machi, Kanazawa, Ishikawa 920-8641 Japan; 7 Department of Respiratory Medicine, University Hospital, Kanazawa University, 13-1 Takara-machi, Kanazawa, Ishikawa 920-8641 Japan; 8 Current affiliation: Department of Pulmonary Medicine and Oncology, Graduate School of Medicine, Nippon Medical School, 1-1-5 Sendagi, Bunkyo-ku, Tokyo, 113-8603 Japan

**Keywords:** Non-small cell lung cancer, Gefitinib, Erlotinib, Hepatotoxicity, BMI, Acid-suppressing medications

## Abstract

**Background:**

Gefitinib and erlotinib, are epidermal growth factor receptor tyrosine kinase inhibitors, and are currently recommended for non-small cell lung cancer stage IV in the elderly and in patients with decreased performance status in the Japanese Lung Cancer Society Guideline, but they occasionally caused severe hepatotoxicity requiring postponement or modification of treatment. However, little is known about the risk factors for hepatotoxicity in patients receiving gefitinib and erlotinib. In this study, we investigated the factors influencing hepatotoxicity in Japanese non-small cell lung cancer (NSCLC) patients treated with gefitinib or erlotinib monotherapy.

**Methods:**

Japanese patients with NSCLC who started gefitinib or erlotinib monotherapy from January 2005 to December 2017 at Kanazawa University Hospital or Kanazawa Medical University Hospital were included in this study. Factors affecting hepatotoxicity were retrospectively investigated by multiple logistic regression analysis.

**Results:**

A total of 102 patients who received gefitinib and 95 patients who received erlotinib were included in the analysis. In the gefitinib group, a body mass index (BMI) ≥ 25 was associated with an increased risk of hepatotoxicity (OR = 4.571, 95% CI = 1.486–14.056, *P* = 0.008). In the erlotinib group, concomitant use of acid-suppressing medications (AS), namely proton pump inhibitors or histamine-2 receptor antagonists, was associated with a reduced risk of hepatotoxicity (OR = 0.341, 95% CI = 0.129–0.900, *P* = 0.030).

**Conclusions:**

BMI ≥ 25 in patients treated with gefitinib increased the risk of hepatotoxicity. In contrast, AS combination with erlotinib reduced the risk of hepatotoxicity. Thus, because different factors influence the risk of hepatotoxicity, monitoring for adverse events should take into account patient background factors and concomitant medications.

## Introduction

Chemotherapy for non-small cell lung cancer (NSCLC) patients includes molecular targeted inhibitors, cell-killing anticancer agents, and immune-checkpoint inhibitors represented by programmed death 1 (PD-1) / programmed death ligand 1 (PD-L1) inhibitors [1]. Among them, epidermal growth factor receptor tyrosine kinase inhibitors (EGFR-TKIs) such as gefitinib and erlotinib significantly prolong progression-free survival (PFS) in EGFR mutation-positive patients as monotherapy, compared with platinum-based combination therapy [2, 3]. Gefitinib and erlotinib are recommended in elderly patients with age ≥ 75 years and in patients with performance status (PS) decreased to ≥2 in The Japanese Lung Cancer Society Guideline for non-small cell lung cancer stage IV [1] because of their efficacy and safety [3–6], although the guidelines recommend osimertinib as first-line therapy in NSCLC patients with exon 19 deletions or L858R point mutations in exon 21 of EGFR [1].

Reported adverse events (AEs) caused by EGFR-TKIs include rash, diarrhea, and hepatotoxicity [7]. These AEs are associated with high exposure, namely increased area under the blood concentration-time curve (AUC) and increased serum trough concentration values [8, 9]. Among them, skin rash and diarrhea are mechanism-based AEs, and can be prevented, or at least prevented from becoming severe, by supportive care using moisturizers, topical steroids, and antidiarrheals [10, 11]. However, although there have been a few in vitro studies on the mechanism of EGFR-TKI-induced hepatotoxicity [12, 13], this has not yet yielded definitive prophylactic treatment or supportive care [14].﻿ Veatch et al. reported that elevated bilirubin and aspartate aminotransferase (AS﻿T) levels of Grade ≥ 3 in patients were associated with increased treatment-related mortality [15]. In addition, Sakata et al. reported that when severe AEs occur, especially hepatotoxicity, switching EGFR-TKIs or temporary drug withdrawal may improve the prognosis of patients with EGFR mutation-positive NSCLC [16].﻿ In a pooled analysis of 21 prospective clinical trials conducted between 2004 and 2014, the incidence of severe hepatotoxicity with elevated AS﻿T﻿ or alanine aminotransferase (ALT) of Grade ≥ 3 on the Common Terminology Criteria for Advanced Events (CTCAE) was 18.0% for gefitinib and 5.4% for erlotinib. Moreover, the incidence of hepatotoxicity with gefitinib was 18.5% in Asians vs. 3.2% in non-Asians [17]. There have been a few previous reports on factors associated with the risk of hepatotoxicity due to gefitinib and erlotinib. Factors identified so far include age < 65, exon 19 deletion mutations in EGFR, concomitant use of acid-suppressing medications (AS), namely proton pump inhibitors (PPIs) and histamine-2 receptor antagonists (H_2_RAs), and body mass index (BMI) ≥ 25 for gefitinib, and age ≥ 65, concomitant use of cytochrome P450 (CYP) 3A4 inducers and AS, and liver metastases for erlotinib [18–20]. However, further study is needed to accumulate evidence that would be helpful in the selection of appropriate EGFR-TKIs in order to minimize the risk of hepatotoxicity in individual patients.

Doses of EGFR-TKIs in a clinical setting are usually fixed according to recommendations in the package inserts without consideration of body size, and therefore differences in dose per body weight might be associated with the occurrence of AEs. Additionally, since the profile of AEs varies among different EGFR-TKIs, knowledge of these profiles is important for selecting the most appropriate EGFR-TKI and providing information to patients [21].

The aim of this study was to clarify risk factors related to hepatotoxicity in NSCLC patients receiving gefitinib or erlotinib monotherapy, currently used in the elderly and in patients with decreased PS according to the guideline [1], focusing on the influence of body size, concomitant medications, pharmacokinetics, and other factors, in order to help provide a rational basis for the management of AEs.

## Materials & methods

Patients with NSCLC who started gefitinib or erlotinib monotherapy at Kanazawa University Hospital or Kanazawa Medical University Hospital between January 2005 and December 2017 were retrospectively studied using their electronic medical records. We excluded patients who started treatment at a reduced dose (less than 250 mg/day for gefitinib and less than 150 mg/day for erlotinib), those who were on concomitant therapy with other anticancer agents, and those with missing data (e.g., weight and height at the start of treatment, laboratory values required for evaluation of factors and hepatotoxicity, or patients who transferred to another hospital). If any of AST, ALT, total bilirubin (T-Bil), or alkaline phosphatase (ALP) as evaluated by the Common Terminology Criteria for Adverse Events (CTCAE) version 5.0 were Grade ≥ 2, or if liver metastasis was present at the time of initiation of treatment, those patients were also excluded. The following data were collected: age, sex, body surface area (BSA), BMI, type of EGFR mutation, smoking history, Eastern Cooperative Oncology Group Performance Status (ECOG PS), stages of cancer, treatment line, presence of metastasis, biochemical parameters, and concomitant medication. Age cutoff was defined based on guidelines and previous reports [1, 4, 6]. BSA was calculated according to the DuBois formula: BSA (m^2^) = [body weight (kg)]^0.425^ × [height (cm)]^0.725^ × 0.007184 [22]. BSA cutoff value was defined based on previous reports [19]. BMI was calculated according to the following equation: BMI (kg / m^2^) = body weight (kg) / height (m) × height (m). BMI cutoff values were set according to the World Health Organization (WHO) classification, with BMI ≥ 25 being overweight [23]. Concomitant medications, defined as a duration of concomitant use of ≥1 week for AS and CYP3A4 inhibitors, and of ≥2 weeks for CYP3A4 inducers [24–26] prior to the occurrence of hepatotoxicity, were investigated. Among AS concomitant medications, omeprazole, lansoprazole, rabeprazole, esomeprazole, and vonoprazan are categorized as PPIs, while cimetidine, famotidine, lafutidine, ranitidine, nizatidine, and roxatidine are categorized as H_2_RAs. CYP3A4 inhibitors include clarithromycin, erythromycin, fluconazole, voriconazole, itraconazole, and verapamil, CYP1A2 inhibitors include amiodarone, ciprofloxacin, and fluvoxamine. CYP3A4 and CYP1A2 inducers include phenobarbital, phenytoin, carbamazepine, and rifampicin. Based on the European Association for the Study of the Liver (EASL) Guidelines for Drug-induced Liver Disorders and previous reports [19, 27], hepatotoxicity was defined as the presence any of AST, ALT, T-Bil, or ALP of grade ≥ 2 as evaluated by CTCAE version 5.0. This clinical study was conducted with the approval of the Ethical Review Committees of Kanazawa University (Approval No. 2017–257) and Kanazawa Medical University (Approval No. H 189).

### Statistical analysis

Patients’ background factors were analyzed by using Fisher’s exact test or the Mann-Whitney U test. Factors with *P* <  0.200 in univariate analysis and those considered of high clinical importance based on previous reports were included in subsequent multiple logistic regression analysis using the forced imputation method. The Kaplan-Meier method was used to analyze the time to first occurrence of hepatotoxicity, and the log-rank test was used to compare factors. All analyses were two-tailed, and *P* <  0.05 was considered statistically significant. Statistical analysis software used was IBM SPSS Statistics 27 (IBM Japan Ltd., Tokyo) or EZR (Saitama Medical Center, Jichi Medical University, Saitama, Japan), which is a graphical user interface for R (The R Foundation for Statistical Computing, Vienna, Austria, version 4.0.3). Specifically, EZR is a modified version of R commander (version 2.7–1), which was designed to add statistical functions frequently used in biostatistics [28].

## Results

### Patients

**Fig. 1:**
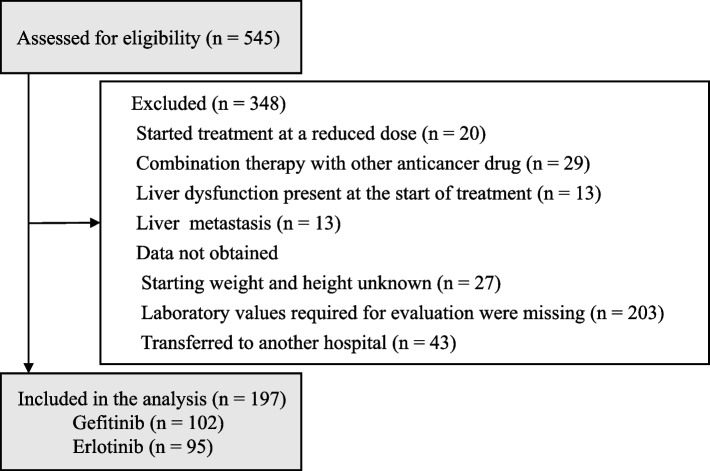
Flow diagram of patient selection. The diagram shows the number of patients enrolled in the study and included in the analysis, as well as the number of patients excluded and the reasons for their exclusion (there may be more than one reason for exclusion). Liver dysfunction is defined as grade 2 or higher in any of AST, ALT, T-Bil, or ALP, as evaluated by CTCAE version 5.0

A total of 545 patients were included in the study. Three hundred forty-eight patients were excluded based on the exclusion criteria, and 197 patients were included in the analysis. Of these 197 patients, 102 received gefitinib, and 95 received erlotinib (Fig. 1), and their characteristics are summarized in Table 1. The variables that showed a significant difference between the two groups were age, age cut-off value of 75 years, BSA, and the treatment line.

**Table 1:** Patients’ characteristics

	No. of patients (%) ^a^		
Variable	Gefitinib	Erlotinib	*P*
	(*n* = 102)	(*n* = 95)	
Age (years)			
Median [range]	73 [37–90]	68 [37–87]	0.001 ^c^
< 75	60 (59)	78 (82)	< 0.001 ^d^
≥ 75	42 (41)	17 (18)	
Sex			
Male	46 (45)	50 (53)	0.180 ^d^
Female	56 (55)	45 (47)	
ECOG PS			
0–1	86 (84)	84 (88)	0.417 ^d^
2–3	16 (16)	11 (12)	
Stage			
I - III	49 (48)	36 (38)	0.195 ^d^
IV	53 (52)	59 (62)	
BSA (m^2^)			
Median [range]	1.5 [1.1–1.9]	1.6 [1.1–2.0]	0.045 ^c^
< 1.6	70 (69)	54 (57)	0.105 ^d^
≥ 1.6	32 (31)	41 (43)	
BMI (kg/m^2^)			
Median [range]	21.7 [14.9–31.3]	22.0 [15.3–30.3]	0.796 ^c^
< BMI 25	83 (81)	81 (85)	0.568 ^d^
≥ BMI 25	19 (19)	14 (15)	
Recurrence			
Yes	41 (40)	34 (36)	0.559 ^d^
No	61 (60)	61 (64)	
Metastasis			
Yes	91 (89)	88 (93)	0.465 ^d^
No	11 (11)	7 (7)	
Treatment line			
First line	59 (58)	26 (27)	< 0.001 ^d^
Second line and later line	43 (42)	69 (73)	
EGFR mutation			
Exon 19 Deletion			
Yes	44 (43)	34 (36)	0.311 ^d^
No	58 (57)	61 (64)	
Exon 21 L858R point mutation			
Yes	51 (50)	38 (40)	0.197 ^d^
No	51 (50)	57 (60)	
Smoking history			
Yes	39 (38)	46 (48)	0.154 ^d^
No	63 (62)	49 (52)	
Grade 1 Liver dysfunction ^b^			
Yes	37 (36)	31 (33)	0.654 ^d^
No	65 (64)	64 (67)	

^a^ Percentages are rounded off

^b^ Any of AST, ALT, T-Bil, or ALP grade 1 as evaluated by CTCAE version 5.0 at the start of treatment

^c^ Mann-Whitney U test

^d^ Fisher’s exact test

### Univariate and multivariate analysis of factors affecting the development of hepatotoxicity with gefitinib

For univariate and multivariate analyses, patients were divided into two groups: those with and without hepatotoxicity. In the univariate analysis, six factors: BMI, BMI ≥ 25, treatment line, mutation of exon 19 deletion in EGFR, smoking history, and concomitant AS showed *P* <  0.200, suggesting a possible association with the occurrence of hepatotoxicity. Furthermore, based on the criteria mentioned in the Statistical Analysis section, BMI ≥ 25, mutation of exon 19 deletion of EGFR, and concomitant use of AS, were entered into the multivariate analysis (Table 2). The results of multivariate analysis showed that BMI ≥ 25 was an independent factor affecting gefitinib-induced hepatotoxicity (OR = 4.571, 95% CI = 1.486–14.056, *P* = 0.008).

**Table 2:** Univariate and multivariate analysis of factors affecting the development of hepatotoxicity with gefitinib

Variable	Univariate analysis		*P*	Multivariate analysis	
	No. of patients (%) ^a^			OR (95% CI)	*P*^f^
	Absence	Presence			
	(*n* = 72)	(*n* = 30)			
Age (years)					
Median [range]	72 [47–90]	72 [37–85]	0.700 ^d^		
< 75	43 (60)	17 (57)	0.827 ^e^		
≥ 75	29 (40)	13 (43)			
Sex					
Male	34 (47)	12 (40)	0.522 ^e^		
Female	38 (53)	18 (60)			
ECOG PS					
0–1	60 (83)	26 (87)	0.773 ^e^		
2–3	12 (17)	4 (13)			
Stage					
I - III	35 (49)	14 (47)	1.000 ^e^		
IV	37 (51)	16 (53)			
BSA (m^2^)					
Median [range]	1.5 [1.1–1.8]	1.5 [1.1–1.9]	0.467 ^d^		
< 1.6	52 (72)	18 (60)	0.248 ^e^		
≥ 1.6	20 (28)	12 (40)			
BMI (kg/m^2^)					
Median [range]	21.1 [14.9–31.3]	23.9 [16.2–28.5]	0.016 ^d^		
< BMI 25	63 (88)	20 (67)	0.024 ^e^	4.571 (1.486–14.056)	0.008
≥ BMI 25	9 (12)	10 (33)			
Recurrence					
Yes	28 (39)	13 (43)	0.825 ^e^		
No	44 (61)	17 (57)			
Metastasis					
Yes	64 (89)	27 (90)	1.000 ^e^		
No	8 (11)	3 (10)			
Line of therapy					
First line	38 (53)	21 (70)	0.127 ^e^		
Second line and later line	34 (47)	9 (30)			
EGFR mutation					
Exon 19 Deletion					
Yes	27 (38)	17 (57)	0.084 ^e^	2.327 (0.918–5.895)	0.075
No	45 (63)	13 (43)			
Exon 21 L858R point mutation					
Yes	38 (53)	13 (43)	0.515 ^e^		
No	34 (47)	17 (57)			
Smoking history					
Yes	32 (44)	7 (23)	0.073 ^e^		
No	40 (56)	23 (77)			
Grade 1 liver dysfunction ^b^					
Yes	24 (33)	13 (43)	0.372 ^e^		
No	48 (67)	17 (57)			
Concomitant medication ^c^					
AS					
Yes	33 (46)	8 (27)	0.081 ^e^	0.394 (0.145–1.070)	0.068
No	39 (54)	22 (73)			
CYP1A2 inducer					
Yes	1 (1.0)	0 (0.0)	1.000 ^e^		
No	71 (99)	30 (100)			
CYP3A4 inducer					
Yes	1 (1.0)	0 (0.0)	1.000 ^e^		
No	71 (99)	30 (100)			
CYP3A4 inhibitor					
Yes	5 (7.0)	0 (0.0)	0.318 ^e^		
No	67 (93)	30 (100)			

^a^ The sum of the percentages may not equal 100% because of rounding off

^b^ Any of AST, ALT, T-Bil, or ALP grade 1 as evaluated by CTCAE version 5.0 at the start of treatment

^c^ Concomitant medications that were being taken at the time of the hepatotoxicity

^d^ Mann-Whitney U test

^e^ Fisher’s exact test

^f^ Logistic regression analysis

*OR* odds ratio, *CI* confidence interval, *ECOG PS* Eastern Cooperative Oncology Group performance status, *BSA *body Surface Area, *BMI* body mass index, *EGFR* epidermal growth factor receptor, *AS* acid-suppressing medications, *CYP* cytochrome P450

### Univariate and multivariate analysis of factors affecting the development of hepatotoxicity with erlotinib

As with gefitinib-treated patients, patients were divided into groups with and without hepatotoxicity. In the univariate analysis, both sex and concomitant use of AS showed *P* <  0.200, suggesting a possible association with the occurrence of hepatotoxicity. These two factors were included in the multivariate analysis (Table 3). The results of multivariate analysis showed that concomitant use of AS was an independent factor reducing the occurrence of hepatotoxicity (OR = 0.341, 95% CI = 0.129–0.900, *P* = 0.030).

**Table 3:** Univariate and multivariate analysis of factors affecting the development of hepatotoxicity with erlotinib

Variable	Univariate analysis		*P*	Multivariate analysis	
	No. of patients (%) ^a^			OR (95% CI)	*P*^f^
	Absence	Presence			
	(*n* = 69)	(*n* = 26)			
Age (years)					
Median [range]	68 [37–87]	68 [55–83]	0.565 ^d^		
< 75	57 (83)	21 (81)	1.000 ^e^		
≥ 75	12 (17)	5 (19)			
Sex					
Male	40 (58)	10 (39)	0.109 ^e^	2.032 (0.787–5.248)	0.143
Female	29 (42)	16 (62)			
ECOG PS					
0–1	60 (87)	24 (92)	0.722 ^e^		
2–3	9 (13)	2 (8.0)			
Stage					
I-III	26 (38)	10 (39)	1.000 ^e^		
IV	43 (62)	16 (62)			
BSA (m^2^)					
Median [range]	1.6 [1.1–2.0]	1.6 [1.3–1.9]	0.478 ^d^		
< 1.6	39 (57)	15 (58)	1.000 ^e^		
≥ 1.6	30 (43)	11 (42)			
BMI (kg/m^2^)					
Median [range]	22.1 [15.3–30.3]	21.4 [16.8–29.1]	0.652 ^d^		
< BMI 25	59 (86)	22 (85)	1.000 ^e^		
≥ BMI 25	10 (15)	4 (15)			
Recurrence					
Yes	23 (33)	11 (42)	0.475 ^e^		
No	46 (67)	15 (58)			
Metastasis					
Yes	64 (93)	24 (92)	1.000 ^e^		
No	5 (7)	2 (8.0)			
Line of therapy					
First line	18 (26)	8 (31)	0.797 ^e^		
Second line and later line	51 (74)	18 (69)			
EGFR mutation					
Exon 19 Deletion					
Yes	24 (35)	10 (39)	0.812 ^e^		
No	45 (65)	16 (62)			
Exon 21 L858R point mutation					
Yes	27 (39)	11 (42)	0.817 ^e^		
No	42 (60)	15 (58)			
Smoking history					
Yes	36 (52)	10 (39)	0.258 ^e^		
No	33 (48)	16 (62)			
Grade 1 liver dysfunction ^b^					
Yes	21 (30)	10 (38)	0.471 ^e^		
No	48 (70)	16 (62)			
Concomitant medication ^c^					
AS					
Yes	40 (58)	8 (31)	0.022 ^e^	0.341 (0.129–0.900)	0.030
No	29 (42)	18 (69)			
CYP1A2 inducer					
Yes	2 (3.0)	0 (0.0)	1.000 ^e^		
No	67 (97)	26 (100)			
CYP3A4 inducer					
Yes	2 (3.0)	0 (0.0)	1.000 ^e^		
No	67 (97)	26 (100)			
CYP3A4 inhibitor					
Yes	5 (7.0)	1 (4.0)	1.000 ^e^		
No	64 (93)	25 (96)			

^a^ The sum of the percentages may not equal 100% because of rounding off

^b^ Any of AST, ALT, T-Bil, or ALP grade 1 as evaluated by CTCAE version 5.0 at the start of treatment

^c^ Concomitant medications that were being taken at the time of the hepatotoxicity

^d^ Mann-Whitney U test

^e^ Fisher’s exact test

^f^ Logistic regression analysis

*OR* odds ratio, *CI* confidence interval, *ECOG PS* Eastern Cooperative Oncology Group performance status, *BSA *body Surface Area, *BMI* body mass index, *EGFR* epidermal growth factor receptor, *AS* acid-suppressing medications, *CYP* cytochrome P450

### Analysis of the association of first onset of hepatotoxicity and BMI in patients treated with gefitinib and erlotinib

**Fig. 2:**
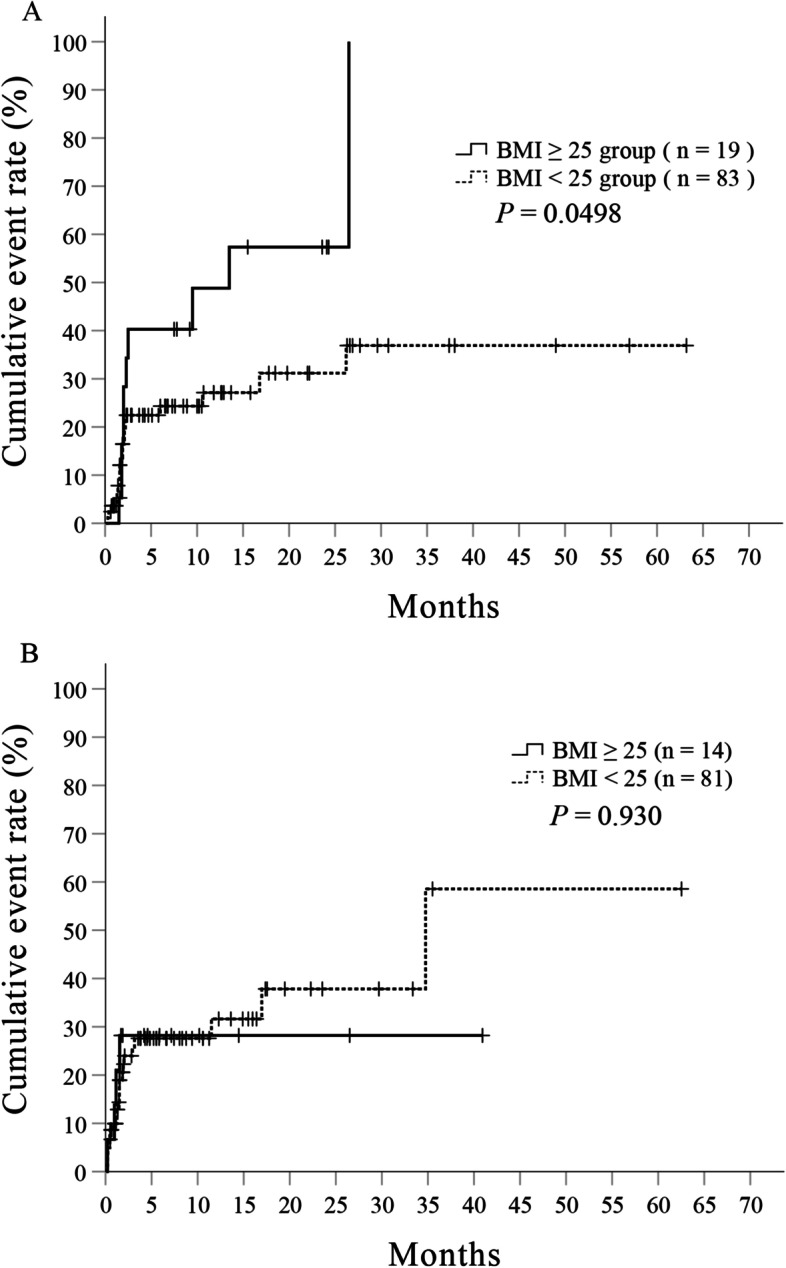
Kaplan-Meier curve of the association between the occurrence of first hepatotoxicity and BMI. Kaplan-Meier curve in gefitinib-treated patients (**A**) and in erlotinib-treated patients (**B**). A log-rank test was used for statistical analysis

In patients treated with gefitinib, the median time to first onset of hepatotoxicity was 13.5 months (95% CI = 1.97 to not applicable) in patients with BMI ≥ 25, and the median time was not reached during the observation period in patients with BMI < 25 (Fig. 2A). The log-rank test showed a significant difference in the time to onset of hepatotoxicity between the two BMI categories (*P* = 0.0498). On the other hand, in patients who received erlotinib, the median time to first onset of hepatotoxicity was not reached during the observation period in patients with BMI ≥ 25, while the median time in patients with BMI < 25 was 34.8 months (95% CI = 17.0 to not applicable), and there was no significant difference (*P* = 0.930) (Fig. 2B).

## Discussion

In this study, multivariate analysis identified BMI ≥ 25 in patients treated with gefitinib and concomitant use of AS in patients treated with erlotinib as independent factors influencing the occurrence of hepatotoxicity.

BMI ≥ 25 in patients with gefitinib was associated with increased risk of hepatotoxicity, and furthermore, patients with BMI ≥ 25 had a shorter time to first onset of hepatotoxicity than those with BMI < 25. In addition, the median BMI was significantly higher in patients who developed hepatotoxicity than in patients who did not. Oda et al. also reported that BMI ≥ 25.0 was a risk factor for the occurrence of hepatotoxicity in patients with EGFR-mutated NSCLC undergoing gefitinib monotherapy [20], in accordance with the present results. In obese patients, it was commonly reported that the activity of CYP3A4 was decreased [29–32], although CYP2D6 and 1A2 were not affected [29, 30], and that the clearance of CYP3A4 substrates fentanyl and amiodarone was decreased [33, 34]. Since gefitinib is metabolized mainly by CYP3A4, and partially by CYP3A5, 2D6, 1A1, and 1A2 [35–37], reduced CYP3A4 activity in obese patients is expected to cause reduced clearance of gefitinib, which would lead to earlier occurrence of hepatotoxicity compared to patients with normal BMI. In contrast, BMI was not a risk factor for erlotinib. One reason for this may be that the contribution of CYP3A4 to metabolism of erlotinib is smaller than that in the case of gefitinib [35, 37, 38]. Thus, the contribution of CYP3A4 to metabolism of EGFR-TKIs may be a key determinant of hepatotoxicity in obese patients. On the other hand, although afatinib is hardly metabolized by CYPs, low BMI, low body weight, low BSA, female sex, and elderly status were risk factors for the occurrence of grade ≥ 3 diarrhea, and this is likely to be related to increased exposure to afatinib in patients with low BMI who receive the standard dose [39, 40]. Thus, the appropriate dosage of EGFR-TKIs appears to be dependent upon not only BMI, but also the type of EGFR-TKI, especially the contribution of CYP3A4 to the metabolism. Clinically, the perspective of considering weight and BMI in risk management for AEs is not well known, and even with gefitinib and erlotinib, dosage adjustment based on body size has not been performed. However, the results of this study, as well as the pattern for afatinib [39, 40], suggest that this would be important for risk management of AEs; especially, more careful liver function monitoring is desirable in patients with low or high BMI when initiating treatment with gefitinib. On the other hand, we could not investigate the influence of nonalcoholic fatty liver disease (NAFLD) and nonalcoholic steatohepatitis (NASH), which are associated with high BMI [41]. NSAH is itself a risk factor for liver dysfunction [41], and further study is needed to investigate the relationship of obesity-associated factors such as CYP3A4 or NAFLD/NASH with hepatotoxicity.

In patients treated with erlotinib, concomitant use of AS decreased the risk of hepatotoxicity. Gefitinib-treated patients also showed a reduced risk of hepatotoxicity in univariate analysis, although there was no statistically significant difference in multivariate analysis. Erlotinib and gefitinib exhibit pH-dependent solubility, and their AUCs decrease when they are used in combination with AS, such as PPIs and H_2_RAs [42–44]. Similar drug-drug interactions between AS and TKIs such as pazopanib and dasatinib have been reported [42–44]. For example, erlotinib showed a 46% decrease in AUC when combined with 40 mg of omeprazole daily, owing to a decrease in drug solubility [42]. Therefore, the reduced risk of hepatotoxicity with erlotinib is considered to be caused by the decrease in bioavailability and exposure due to concomitant use of AS. However, there is a conflicting report that concomitant use of PPIs or H_2_RAs increased the risk of hepatotoxicity in patients receiving erlotinib and gefitinib, and it was speculated that the mechanism might involve drug-drug interaction at ATP-binding cassette subfamily G member 2 (ABCG2) [18, 19]. Indeed, erlotinib and gefitinib are substrates of ABCG2, and PPIs inhibit ABCG2. However, the half-maximal inhibitory concentration (IC_50_) values of PPIs for ABCG2 in vitro are 50–200 times higher than the unbound concentration at the clinical dose [45], which suggests that the contribution of interaction between EGFR-TKIs and AS at ABCG2 might be negligible in the present study. From the viewpoint of clinical outcome, it has been reported that the concomitant use of EGFR-TKI and AS was associated with reduced OS and PFS [46, 47], but on the other hand, there are conflicting reports that concomitant use of AS did not affect OS and PFS [48, 49]. Kumarakulasinghe et al. considered that one of the reasons for the apparent discrepancies might be high sensitivity of EGFR-TKIs to EGFR mutation positivity [49]. In addition, various other factors affect OS and PFS, including the patient’s PS, metastasis to other organs, and treatment strategy [46–49]. Since OS and PFS could not be evaluated in this study, further work will be needed to clarify the effects of AS on OS and PFS as independent factors. It is desirable in clinical practice to evaluate the need for concomitant use of AS in individual patients depending upon the type of TKIs, with due consideration of its impact on efficacy. In the case of erlotinib, the risk of hepatotoxicity may fluctuate with concomitant use of AS, considering the results of this study and previous reports [19]. Therefore, we suggest that more attention be paid to liver function trends, especially when concomitant use of AS is initiated or discontinued.

Other reported risk factors for hepatotoxicity include age < 65 and exon 19 deletion mutations in EGFR for gefitinib and age ≥ 65 and concomitant use of CYP3A4 inducers for erlotinib [18, 19]. In patients with exon 19 deletion mutation of EGFR, PFS was significantly prolonged compared with cell-killing chemotherapy [2], and hepatotoxicity has been reported to increase with prolonged treatment [50]. In the gefitinib group in this study, exon 19 deletion mutation of EGFR was also found to be a significant risk factor for hepatotoxicity in univariate analysis, suggesting that the difference in the treatment duration was a confounding factor. Regarding age, generally, drug AEs are more frequently observed in elderly patients receiving polypharmacy or who have organ dysfunctions. On the other hand, some reports have described high efficacy and safety of erlotinib and gefitinib in elderly patients [4–6]. In an analysis of 9907 Japanese patients treated with erlotinib (2059 were age ≥ 75 years), the incidence of AEs, including hepatotoxicity, in elderly patients was similar to that in younger patients [6]. In the present study, we also examined age, but there was no significant difference. The concomitant use of CYP3A4 inducer was reported as a risk factor for hepatotoxicity with erlotinib, and it was suggested that CYP3A4-mediated active metabolite formation was involved. However, the results were obtained for a mixed population of NSCLC and pancreatic cancer, and risk factor analysis for each cancer type was not conducted. Another reason for the inconsistency may be the small number of cases in our study. On the other hand, it is generally considered that concomitant use of CYP3A4 inducers decreases the blood concentrations of many drugs [51–53], including those that cause hepatotoxicity, such as gefitinib and erlotinib.

There are several limitations in this study. First, we were unable to evaluate the blood concentration of the drugs, because this was a retrospective study. Future prospective studies will be needed to clarify the effects of BMI and AS on the pharmacokinetics of gefitinib and erlotinib and their contribution to hepatotoxicity. Secondly, since the mechanism of hepatotoxicity caused by gefitinib and erlotinib was not clear, we defined hepatotoxicity using AST, ALT, T-Bil, or ALP according to the published guidelines, which is a different definition compared to previous reports. Therefore, we performed a sensitivity analysis based on the use of only AST or ALT as a criterion of hepatotoxicity. Similar results were obtained in the gefitinib group (*n* = 26), i.e., that BMI ≥ 25 is a significant risk factor (*P* = 0.003) for hepatotoxicity. On the other hand, a similar trend could not be identified in the erlotinib group (*n* = 9), due to the small number of cases. Thirdly, concomitant medications and comorbidities may not have been adequately considered. In particular, the number of cases in which inducers or inhibitors of CYP3A4 and CYP1A2 were used was very small in our study, making it difficult to assess the risk of concomitant use. In addition, since the interaction of H_2_RA should be attenuated by separation of the timing of dosing of H_2_RA and EGFR-TKIs [42], evaluation of the appropriate dosing timing will also be necessary to clarify the effect of H_2_RA on the pharmacokinetics of EGFR-TKIs. Regarding comorbidities, we could not evaluate the effect of liver diseases, such as NAFLD/NASH in particular, which may have augmented the hepatotoxicity caused by TKIs. Finally, the sample size was small in our study, and there may have been some bias in the patients’ background factors.

According to the guideline [1], gefitinib and erlotinib are currently recommended for elderly patients and patients with decreased PS who are vulnerable and especially those who require intensive monitoring. In particular, careful management is needed to prevent severe disease in patients with a high BMI and when starting or discontinuing concomitant use of AS, as these were identified as risk factors of hepatotoxicity in this study. Further studies will be needed to examine the risk of AEs from newer drugs, as well as the role of other background factors.

## Conclusion

We identified independent factors that influence the hepatotoxicity of gefitinib and erlotinib. BMI ≥ 25 increased the risk of hepatotoxicity in gefitinib monotherapy, and concomitant use of AS reduced the risk of hepatotoxicity in erlotinib monotherapy. Since different factors influence the risk of hepatotoxicity, our findings may be useful for assessing and managing the safety of continued treatment based on individual patient backgrounds and concomitant medications.

## Data Availability

All data generated or analyzed during this study are included in this published article.

## Abbreviations

ABCG2: ATP-binding cassette subfamily G member 2
AEs: Adverse events
ALP: Alkaline phosphatase
ALT: Alanine aminotransferase
AS: Acid-suppressing medications
AST: Aspartate aminotransferase
AUC: Area under the blood concentration-time curve
BMI: Body mass index
BSA: Body surface area
CI: Confidence interval
CTCAE: Common Terminology Criteria for Advanced Events
CYP: Cytochrome P450
ECOG PS: Eastern Cooperative Oncology Group Performance Status
EGFR-TKIs: Epidermal growth factor receptor tyrosine kinase inhibitors
H_2_RAs: Histamine-2 receptor antagonists
IC_50_: Half-maximal inhibitory concentration
NAFLD: Nonalcoholic fatty liver disease
NASH: Nonalcoholic steatohepatitis
NSCLC: Non-small cell lung cancer
OR: Odds ratio
PD-1: Programmed death 1
PD-L1: Programmed death ligand 1
PFS: Progression-free survival
PPIs: Proton pump inhibitors
PS: Performance status
T-Bil: Total bilirubin
WHO: World Health Organization
